# Swine influenza A (H1N1) virus (SIV) infection requiring extracorporeal life support in an immunocompetent adult patient with indirect exposure to pigs, Italy, October 2016

**DOI:** 10.2807/1560-7917.ES.2017.22.5.30456

**Published:** 2017-02-02

**Authors:** Francesca Rovida, Antonio Piralla, Federico Capra Marzani, Ana Moreno, Giulia Campanini, Francesco Mojoli, Marco Pozzi, Alessia Girello, Chiara Chiapponi, Fausto Vezzoli, Paola Prati, Elena Percivalle, Anna Pavan, Maria Gramegna, Giorgio Antonio Iotti, Fausto Baldanti

**Affiliations:** 1 SS Virologia Molecolare, SC Microbiologia e Virologia, Fondazione IRCCS Policlinico San Matteo, Pavia, Italy; 2 These authors contributed equally to this work; 3 Anestesia e Rianimazione, Dipartimento di Emergenza ed Urgenza, Fondazione IRCCS Policlinico S. Matteo, Pavia, Italy; 4 Istituto Zooprofilattico Sperimentale della Lombardia ed Emilia Romagna, Brescia, Italy; 5 Unità di Anestesia, Rianimazione e Terapia Antalgica, Dipartimento di Scienze Clinico-Chirurgiche, Diagnostiche e Pediatriche, Università degli Studi di Pavia, Pavia, Italy; 6 Istituto Zooprofilattico Sperimentale della Lombardia ed Emilia Romagna, Parma, Italy; 7 Istituto Zooprofilattico Sperimentale della Lombardia ed Emilia Romagna, Lodi, Italy; 8 Istituto Zooprofilattico Sperimentale della Lombardia ed Emilia Romagna, Pavia, Italy; 9 Agenzia di Tutela della Salute, Pavia, Italy; 10 Direzione Generale Sanità, Regione Lombardia, Milan, Italy; 11 Dipartimento di Scienze Clinico-Chirurgiche, Diagnostiche e Pediatriche, Università degli Studi di Pavia, Pavia, Italy

**Keywords:** influenza virus, respiratory infection, extracorporeal membrane oxygenation

## Abstract

We describe a case of severe swine influenza A(H1N1) virus infection in an immunocompetent middle-aged man in October 2016 in Italy who had only indirect exposure to pigs. The patient developed a severe acute distress respiratory syndrome which was successfully supported by extracorporeal membrane oxygenation and treated with antiviral therapy. The sole risk factor for influenza was a body mass index > 30 kg/m^2^. After a month of hospitalisation, the patient was discharged in good health.

## Case description

**Figure 1:**
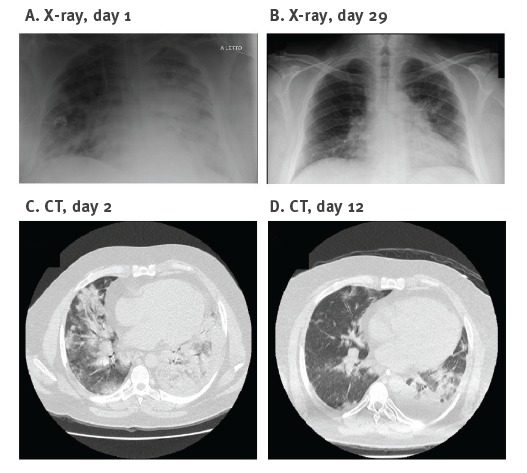
Chest X-ray and computed tomography in a patient with severe swine influenza A(H1N1), Italy, October 2016

In early October 2016, a man in his 40s with underlying obesity (body mass index > 30 kg/m^2^) presented at the emergency department of our hospital after four days of rhinitis, cough, fever and dyspnoea. The patient was hospitalised due to hypoxaemia (P_a_O_2_/F_I_O_2_ = 190), hypocapnia, hyperlactataemia (3.6 mmol/L), dyspnoea and bilateral interstitial pneumonia, as shown by chest X-ray and computed tomography (CT) (Figure 1).

CT: computed tomography.

The X-ray taken on admission (A) showed bilateral opacities. A follow-up chest X-ray performed on day 29 showed almost complete regression of the opacities (B). Chest CT on day 2 after admission (C) showed extensive bilateral consolidation with alveolar parenchymal consolidations and ground-glass opacity (right end). CT on day 12 after admission showed partial regression of consolidation in the right median-lower lobe with residual little areas of ground-glass opacity and improved ventilation of the lower left lobe (D).

On the following day, the patient’s clinical condition worsened (P_a_O_2_/F_I_O_2_ = 65) and he was transferred to the intensive care unit (ICU) with severe acute distress respiratory syndrome (ARDS). The CT scan at ICU admission is shown in Figure 1. The patient was first supported with helmet continuous positive airways pressure (CPAP) and then with invasive mechanical ventilation. On the same day, a nasal swab sample and a bronchoalveolar lavage (BAL) were collected and tested by real-time RT-PCR and PCR for a panel of 12 respiratory viruses [1,2]. Influenza A virus was detected, with high viral load in the BAL (9.7 × 10^7^ RNA copies/mL) and a lower viral load in the nasal swab (4.5 × 10^2^ RNA copies/mL), while the results for the remaining 11 viruses were negative. The BAL was negative for the most common bacteria and fungi by standard cultures. Oseltamivir treatment (75 mg twice a day) was started. 

Three days after admission, low-flow veno-venous extracorporeal membrane oxygenation (ECMO) was initiated in order to allow hyperprotective mechanical ventilation with low tidal volume (< 4 mL/kg ideal body weight, which was calculated to be 65 kg). Eight days after admission, respiratory conditions improved and the patient was disconnected from ECMO. The influenza A virus RNA load had decreased considerably (2.0 × 10^4^ copies/mL in BAL and 0 RNA copies/mL in the nasal swab). Five days later, the BAL was negative for influenza A virus RNA but the patient experienced a super-infection with meticillin-resistant *Staphylococcus aureus* and *Pseudomonas aeruginosa,* and therapy with linezolid and piperacillin/tazobactam was administrated. After disconnection from ECMO support, the patient was gradually weaned from mechanical ventilation and subsequently from CPAP. 

After 16 days of ICU, as the patient’s clinical condition was improving, he was transferred to the pneumology ward and after 30 days of overall hospital stay discharged in good health.

## Virological findings

Molecular subtyping of the influenza A strain (A/Pavia/65/2016) was unsuccessful using real-time RT-PCRs specific for human influenza subtypes H1 and H3 directly on biological samples. On day 7 of admission, partial nucleotide sequences of the nucleoprotein and non-structural genes, obtained in an RT-PCR that amplifies all eight segments of the influenza A genome [3], showed that the A/Pavia/65/2016 strain was an influenza A(H1N1) virus of swine origin. The A/Pavia/65/2016 strain was propagated in embryonated specific pathogen–free chicken eggs using BAL and swab samples as inoculum and all eight genome segments were sequenced using the MiSeq platform (Illumina, San Diego, US) as previously described [4] (GenBank accession numbers KY368147–154). The data were de novo assembled on BaseSpace Cloud (Illumina, San Diego, US) with the DNAStar application and analysed with the Lasergene package software (version 10.1.2). 

**Figure 2:**
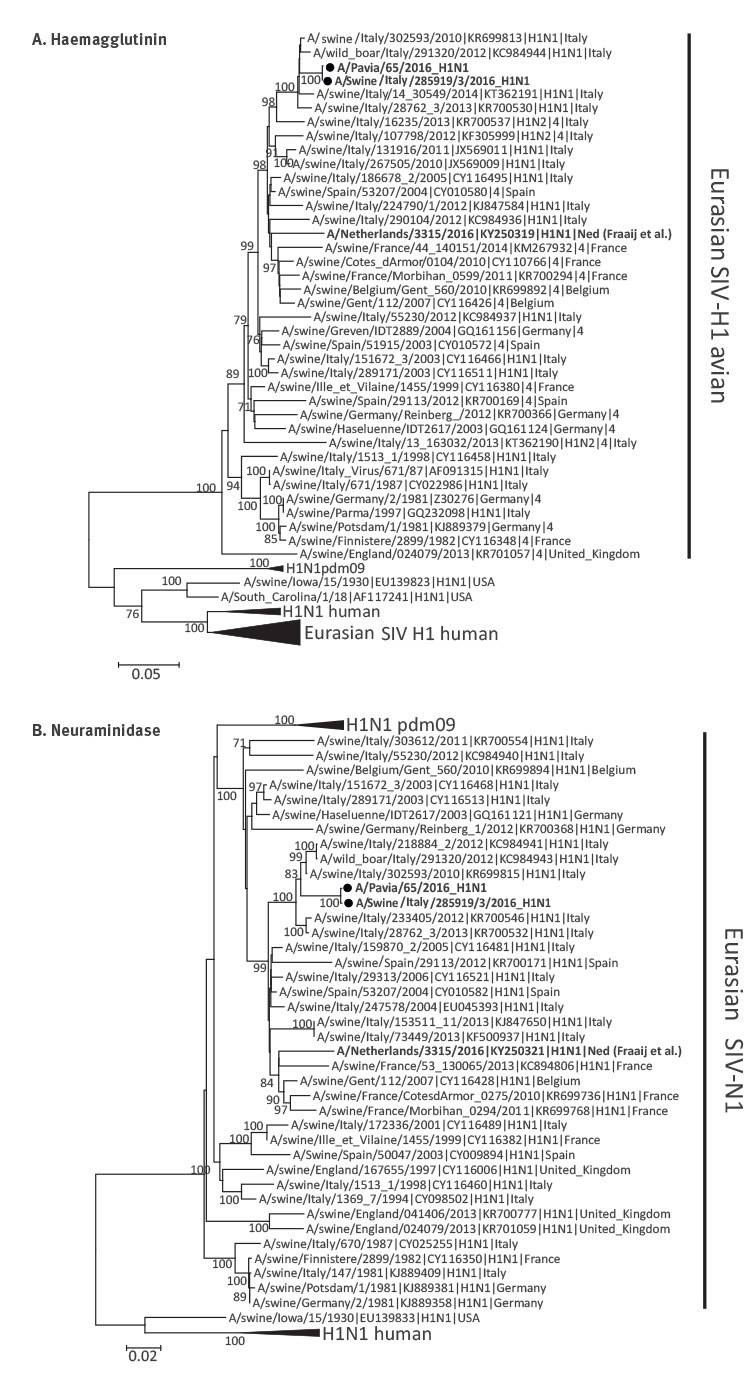
**Phylogenetic relationship of human and swine influenza A(H1N1) virus strains based on complete haemagglutinin and neuraminidase nucleotide sequences, compared with patient isolate, Italy, October 2016**

Phylogenetic analyses were performed using MEGA6 software [5]. A phylogenetic tree of the haemagglutinin (HA) and neuroaminidase (NA) genes confirmed that the A/Pavia/65/2016 strain was closely related to the European avian-like swine influenza A(H1N1) virus (Figure 2). The phylogenetic analysis of the internal genes excluded genome reassortments and showed that all eight segments derived from the Eurasian avian-like lineage.

SIV: swine influenza virus.

The unrooted trees were created by neighbour-joining method implemented in the MEGA 6.0 software and bootstrapped with 1,000 replicates. The pairwise distances were computed using the Kimura 2-parameter model. Only bootstrap values higher than 70% are shown. Patient and pig haemagglutinin and neuraminidase sequences from our study are marked with a black circle and bold letters. Scale bars indicate nucleotide substitutions per site. Sequence reported in Fraaij et al. [6] are highlighted in bold. The other sequences used in the phylogenetic trees were obtained from Watson et al. [12] as well as from the GenBank database.

The patient had not had any direct contact with pigs but lived with a brother employed as a breeder on a pig farm. When interviewed, the patient’s brother reported having had mild respiratory symptoms, in late September to early October. Virological investigations on the pig farm mid-October showed an absence of clinical signs in the animals, but nasal swabs collected from twelve weaning pigs resulted positive by PCR for influenza A, although at a low viral load. Partial genome sequencing of one of the strains (A/swine/Italy/285919/3/2016) included complete HA, NA, M, NS genes and partial PB2, PB1, PA and NP segments (GenBank accession numbers KY368155–162) and proved its close relationship with the A/Pavia/65/2016 strain (Figure 2 and Table), placing it among the Eurasian swine influenza virus (SIV) avian-like strains circulating in Italy.

**Table:** Nucleotide identity of the eight genome segments of the influenza A/Pavia/65/2016 from the patient and the influenza A/swine/Italy/285919/3/2016 (Sw16) obtained on the pig farm vs reference strains, Italy, October 2016

Gene	Influenza A(H1N1) strain				
	Sw16	Sw10	SwNed16	Nc99	Ca09
PB2	99.9^a^	98.3	91.2	81.7	83.5
PB1	99.8^a^	98.2	92.7	80.6	86.3
PA	99.8^a^	98.1	94.0	80.6	81.4
HA	99.9	97.7	91.1	67.3	66.0
NP	98.6^a^	98.5	94.7	78.8	80.4
NA	99.9	97.3	91.8	74.2	88.5
M	100.0	92.6	93.9	83.0	92.0
NS	100.0	99.6	91.8	81.8	76.5

Sw16: Eurasian swine influenza virus A/swine/Italy/285919/3/2016; Sw10: Eurasian swine influenza virus A/swine/Italy/302593/2010 (H1N1); SwNed16: severe swine influenza A(H1N1) case A/Netherlands/3315/2016(H1N1) detected by Fraaij et al. [11]; Nc99: human influenza virus A/New Caledonia/20/1999 (H1N1); Ca99: human influenza virus A/California/07/2009 (H1N1pdm).

GenBank accession numbers represent sequences from the eight segments of influenza A virus: Sw16 (KY368155–162), Sw10 (KR699810–817), SwNed16 (KY250316–323), Nc99 (AY289929, CY033623–629); Ca09 (NC026431–438).

^a ^Nucleotide comparisons were performed with partial sequences of four of the eight genomic segments (PB2: 1,372 nt; PB1: 455 nt; PA: 539 nt; NP: 247 nt).

Genetic distance analysis showed that the A/Pavia/65/2016 strain shared 98.6–100.0% nucleotide identity with the A/swine/Italy/285919/3/2016 strain (Table) and 97.3–99.6% with the Eurasian SIV strain circulating in Italy during 2010, while a lower nucleotide identity (91.1–94.7%) was observed with the SIV strain (A/Netherlands/3315/2016 H1N1) recently detected in an ICU patient in the Netherlands [6].

To confirm the indirect exposure of the patient to the swine influenza A strain, a serum sample of the patient’s brother was tested by haemagglutination-inhibition (HI) test [7]. To remove potential nonspecific inhibitors, human serum was heat-inactivated, adsorbed with chicken red blood cells and treated with receptor-destroying enzyme. The HI assay was performed using the A/Pavia/65/2016 and two reference SIV: A/swine/CA/3633/84 H3N2 and A/swine/Finistere/2899/82. Two-fold serum dilutions were tested starting at 1:10 and showed in the serum sample of the patient’s brother higher antibody titres against the A/Pavia/65/2016 and H3N2 (A/swine/CA/3633/84) strains (1:320) than against the H1N1 (A/swine/Finistere/2899/82) strain (1:20).

## Background

Since the first isolation of a SIV from a human in 1974 [8], sporadic human cases of SIV have been reported in the United States, Canada, Europe and Asia [9-11]. Although most cases of SIV in humans are associated with mild respiratory syndromes [8-11], a case of severe SIV has recently been reported in the Netherlands [6]. Exposure to pigs is often considered a risk factor for human SIV infections [9] and seroepidemiological studies have demonstrated increased rates of SIV infection in occupationally exposed humans [8-10]. People with exposure to swine may be the first to be infected in the event of a novel virus becoming epizootic in a swine herd, and those who work with swine may operate as a bridge for transmission of the virus to their communities [9].

## Discussion

Exposure to swine is often considered a risk factor for human SIV infections [8]. Here we describe a severe case of swine influenza A(H1N1) virus infection requiring ECMO in an adult immunocompetent man with a BMI > 30 as a risk factor, who had indirect exposure to pigs through a brother working as a breeder on a pig farm. Twelve pigs of the farm tested positive for influenza A, and the strain sequenced from one of them was closely related to the virus recovered from the patient. In addition, antibodies against swine influenza A strains (including the strain recovered from the patient) were detected in the serum sample of the patient’s brother. These data support the hypothesis that SIV infecting our patient was circulating on the pig farm and the patient’s brother might have operated as a bridge for transmission of the virus. 

As SIV infection in humans is mild in most cases, its frequency might be underdiagnosed [9,11]. Nevertheless, in the same month of October, Fraaij et al. reported a case of severe infection caused by swine influenza A(H1N1) in a child requiring ECMO support in the Netherlands [6]. In our phylogenetic analysis of HA and NA, this Dutch strain [6] and the strain A/Pavia/65/2016, circulating in the same period in Europe, clustered into distinct branches of the trees. 

## Conclusion

We have reported here a case of severe swine influenza A infection following indirect exposure to pigs. One possible path of infection could be human-to-human. However, other routes (e.g. contact with contaminated clothing, surfaces etc.) cannot be excluded. These data further highlight the need of strict surveillance of influenza in humans and in animals.
